# Association of Hematological and Biochemical Parameters with Serological Markers of Acute Dengue Infection during the 2022 Dengue Outbreak in Nepal

**DOI:** 10.1155/2023/2904422

**Published:** 2023-02-23

**Authors:** Bibek Raj Bhattarai, Abhishek Mishra, Suraj Aryal, Mandira Chhusyabaga, Rajshree Bhujel

**Affiliations:** ^1^Department of Clinical Pathology, Nepal Lab House, Kathmandu, Nepal; ^2^Department of Laboratory Medicine, Manmohan Memorial Institute of Health Sciences, Tribhuvan University, Kathmandu, Nepal; ^3^Tribhuvan University Teaching Hospital, Maharajgunj, Kathmandu, Nepal; ^4^Department of Microbiology, Chitwan Medical College, Tribhuvan University, Bharatpur, Nepal; ^5^Department of Microbiology, School of Health and Allied Sciences, Pokhara University, Pokhara, Nepal; ^6^Central Department of Microbiology, Tribhuvan University, Kirtipur, Nepal

## Abstract

**Background:**

Nepal faced a major dengue outbreak in 2022. The majority of hospitals and laboratories had limited resources for dengue confirmation and had to rely on rapid dengue diagnostic tests. The purpose of the study is to find the predictive hematological and biochemical parameters in each serological phase of dengue infection (NS1 and IgM) that may assist in dengue diagnosis, severity assessment, and patient management via the use of rapid serological tests.

**Method:**

A laboratory-based cross-sectional study was conducted among dengue patients. Rapid antigen (NS1) and serological test (IgM/IgG) was performed to diagnose positive dengue cases. Furthermore, hematological and biochemical investigations were carried out and compared between NS1 and/or IgM-positive participants. A logistic regression analysis was used to identify the validity of the hematological and biochemical characteristics for dengue diagnosis as well as patient management. Receiver-operating characteristic (ROC) curve analysis was used to define the best cut-off, sensitivity, and specificity.

**Result:**

Multiple logistic regression showed thrombocytopenia (OR_A_ = 1.000; *p* = 0.006), leukopenia (OR_A_ = 0.999; *p* < 0.001), glucose level (OR_A_ = 1.028; *p* = 0.029), aspartate aminotransferase (OR_A_ = 1.131; *p* = 0.001), and monocytosis (OR_A_ = 2.332; *p* = 0.020) as significant parameters in the NS1-only positive group. Similarly, thrombocytopenia (OR_A_ = 1.000; *p* = 0.001), glucose level (OR_A_ = 1.037; *p* = 0.004), and aspartate aminotransferase (OR_A_ = 1.141; *p* < 0.001) were significant in IgM-only positive patients. Moreover, thrombocytopenia (OR_A_ = 1.000; *p* < 0.001), leukopenia (OR_A_ = 0.999; *p* < 0.001), glucose (OR_A_ = 1.031; *p* = 0.017), aspartate aminotransferase (OR_A_ = 1.136; *p* < 0.001), and lymphopenia (OR_A_ = 0.520; *p* = 0.067) were independent predictors in both NS1 + IgM positive groups. Platelets consistently demonstrated a higher area under the curve with increased sensitivity and specificity throughout all models, while aspartate aminotransferase (AUC = 0.811) and glucose (AUC = 0.712) demonstrated better results when single IgM positivity was observed. The total leukocyte count performed better when both NS1 + IgM were positive (AUC = 0.814).

**Conclusion:**

Hence, thrombocytopenia, elevated AST, high glucose level, leukopenia with monocytosis, and leukopenia with lymphopenia may predict dengue diagnosis and its severity during an active infection. Therefore, these laboratory parameters can be used to complement less sensitive rapid tests, improve dengue diagnosis, and help with proper patient management.

## 1. Introduction

Dengue virus (DENV) is a 50 nm, single-stranded RNA virus with a genome approximately 11 kb in length [1]. The virus contains three structural genes encoding capsid protein (C), membrane protein (M), and envelope protein (E), as well as seven nonstructural (NS) genes encoding NS1, NS2A, NS2B, NS3, NS4A, NS4B, and NS5 proteins [2]. Dengue virus is transmitted primarily by the vectors *Aedes aegypti* and *Aedes albopictus* and is most prevalent in tropical and subtropical areas. Dengue infection is usually asymptomatic and self-curable [3]. The World Health Organization (WHO) classified symptomatic dengue as dengue with or without warning signs and severe dengue [4]. The incubation period of the virus ranges from 3 to 10 days, typically 5–7 days, and follows a clinical course as a biphasic febrile phase lasting 2–7 days, a critical phase which lasts 24–48 hours, and a convalescent phase [5]. With an estimated infection of about 400 million people annually, the disease now affects more than 100 countries, most of them in Asia, with a disease burden of 70% [3]. Nepal recently had a dengue outbreak in 2022, and as of December 11, 2022, the total cases of dengue had reached 54,232, with 67 deaths [6].

Various diagnostic methods, such as virus-specific serological tests, molecular detection, and virus isolation, are used for the definitive diagnosis of DENV detection [7]. The three markers most commonly used in serological tests are NS1-Ag and IgM for acute infections and IgG for previous infections. NS1-Ag can be detected from the first 0–9 days of symptoms onset, while IgM is detected 4–5 days after symptoms onset, and production may continue approximately for 3 months or more postonset; IgG levels can be detected throughout the life, starting from 10 to 14 days of postinfection [8, 9].

The gold standard test, like nucleic acid amplification tests, is not readily available in hospitals and clinics in a resource-limited country like Nepal. Thus, many of these facilities rely on lateral flow assays (LFA) or immunochromatography (ICT)-based detection methods for dengue diagnosis. Lateral flow assays are friendly to use and have a rapid turnaround time. Detection of NS1-Ag can be as sensitive as a molecular test during the first 0–7 days of onset of symptoms; however, detection can be compromised in secondary infection due to IgG antibodies from a previous infection [9, 10].

Active dengue detection via ICT, while user-friendly, easy to use, and with rapid turnover time, has low sensitivity and low specificity, as well as higher crossreactivity leading to more false positives. Thus, hematological and biochemical parameters can be beneficial as a supportive test for dengue diagnosis in addition to rapid dengue tests via ICT methods. Furthermore, only a few studies have compared and associated the laboratory parameters with the serological markers of dengue, analyzing just the surface. Therefore, this study attempts to provide an in-depth analysis of different hematological and biochemical tests and associate each parameter with a serological marker (NS1 and/or IgM) of acute dengue infection. Thus, the incorporation of biochemical and hematological parameters may act as a supportive parameter for its diagnosis and would be essential for proper patient management to prevent the life-threatening consequences of dengue.

## 2. Methods

### 2.1. Inclusion and Exclusion Criteria

After obtaining written informed consent, participants with fever/body pain along with a positive dengue profile test were included in the study. DENV-infected patients were categorized into NS1-only, IgM-only, and dual positive/both NS1 + IgM-positive groups. Study populations with negative dengue profile tests and abnormal hematological and biochemical profiles were excluded from the study. Patients with positive IgG in the dengue profile test were also excluded. Participants showing no symptoms and further tested negative for dengue profiles along with normal hematological and biochemical parameters were taken as a control group.

### 2.2. Specimen Collection and Processing

Following standard operating procedures, venous blood samples were collected. Whole blood was collected in a K_3_ EDTA vacuum tube and a gel and clot activator tube. A complete blood profile (hemoglobin, RBC and RBC indices, hematocrit, total leukocyte count, differential leukocyte count, and platelets) was performed from blood samples collected in a K_3_ EDTA tube with a hematology analyzer (Beckman Coulter DxH 520, USA). Similarly, a biochemistry analyzer (Selectra Pro S, ELITech Group, Netherlands) was used to perform biochemical analyses on enzymes (ALP, ALT, AST), bilirubin (total and direct), proteins (total protein and albumin), and nonprotein nitrogenous compounds (urea and creatinine) via a serum sample. Neutrophil:lymphocyte ratio (NLR), lymphocyte:monocyte ratio (LMR), and AST/ALT ratio were calculated based on data.

A serum sample was used to detect dengue infection. Qualitative dengue detection was based on the principle of the rapid chromatographic immunoassay (Dengue NS1 + IgM/IgG Combo Rapid Test, Healgen®). Patients with positive dengue cases were tested for either NS1 or IgM positivity or both NS1 and IgM positivity. Any result that was negative on any one of these profiles was treated as a dengue-negative case. All results were verified by a medical laboratory technologist and a microbiologist.

### 2.3. Statistical Analysis

The data were analyzed using IBM SPSS version 25. Shapiro–Wilk normality test was applied to analyze the data for normal distribution. Categorical variable were described as in numbers and percentage. Continuous variables were shown as the median (*Q*_3_−*Q*_1_). Univariate analysis was performed appropriately using the Mann–Whitney *U* test, which was used for overall analysis between dengue positive and dengue negative groups. Likewise, Kruskal Wallis H test, an omnibus test statistic was used to compare > 2 groups. Furthermore, in the case of statistical association, pairwise analysis was performed via Dunn's post hoc test with Bonferroni adjustment, and a *p* value <0.05 was considered significant. Parameters that were mutually significant in both the univariate analysis and the comparative analysis were included in a univariate logistic regression analysis where a *p* value <0.25 was considered significant. In the multivariate logistic regression, a few parameters were added despite insignificant results in univariate logistics due to their clinical relevance. Binary logistic regression (in a dichotomous outcome) and multinomial logistic regression (more than 2 outcomes) were performed as required. Results were presented as crude and adjusted odds ratios with a 95% confidence interval (95% CI). Those variables that yielded the lowest *p* value <0.05 in multivariate logistics have been considered statistically significant. The covariates, which are common in both binary and multinomial logistic regression, were considered true supportive parameters in dengue diagnosis; thus, they were further analyzed for optimum cut-off via maximizing both sensitivity and specificity using the ROC curve.

## 3. Results

### 3.1. Characteristics and Demographics of the Study Population

Out of 348 total study populations, 50.9% (*n* = 177) were dengue positive and 49.1% (*n* = 171) were dengue negative. The median age of overall participants was 33 years (*Q*_3_−*Q*_1_ = 50 years −23 years); participants were predominately male, 58.6% (*n* = 204) vs. female participants, 41.4% (*n* = 144). Among 177 dengue-positive subjects, 23.9% (*n* = 83) were NS1-only positive, 13.8% (*n* = 48) were IgM-only positive, and 13.2% (*n* = 46) were both NS1 + IgM positive. Similarly, 62.7% (*n* = 111) men and 37.3% (*n* = 66) women were dengue positive. Furthermore, the age group 20–29 years was found to have more positive cases (Supplementary Figure 1).

The Mann–Whitney test revealed that the age in the dengue positive group (median = 37 years) was significantly higher than in the dengue negative group (median = 30 years), *U* = 17719.5, *p* < 0.006. Likewise, the Kruskal-Wallis test also showed the significance of age, *H* = 7.949, *p* < 0.047. On the contrary, gender showed no statistical significance in both Mann–Whitney tests, *U* = 13873.5, *p* < 0.115, and the Kruskal-Wallis test, *H* = 3.761, *p* < 0.288, between the dengue positive and dengue negative groups.

### 3.2. Association of Dengue Infection with Laboratory Findings

The overall association of laboratory findings between the dengue positive and negative groups is presented in Table 1. Likewise, the comparison of laboratory findings between NS1 only, IgM only, and both NS1 + IgM positive dengue patients was shown as median (*Q*_3_−*Q*_1_) in Table 2 and the subsequent significance of the Kruskal–Wallis H test and Dunn's post hoc test for pairwise comparison is shown in Table 3. Briefly, in both the Mann–Whitney *U* test and the Kruskal-Wallis H test, low MCV, high MCHC, decreased platelet count, decrease in TLC, high monocyte count, low LMR, increased glucose level, increased total protein, decreased albumin, increased liver enzymes (AST, ALT, and ALP), and an increased AST/ALT ratio were observed in the dengue positive group.

### 3.3. Logistic Regression and Predictive Markers

A logistic regression analysis was performed, with all the significant variables included in the univariate analysis to adjust for confounders and assess the association between the predictors and outcome. Despite the significance of MCV and MCH, they were not further used for logistic regression due to the high correlation (≥0.7) between each other. Neutrophils and lymphocytes were included in multivariate logistics because of their clinical relevance.

Binary logistic regression was used to assess the association between continuous independent laboratory parameters and dichotomous outcomes (dengue positive and dengue negative). Analysis of the overall model showed statistical significance (*χ*^2^ = 363.93, *p* < 0.05) and a percentage accuracy classification (PAC) of 93.7%. Independent variables, TLC (*p*=0.002, OR: 1.000, 95% CI: 0.999–1.000), platelets (*p* < 0.001, OR: 1.000, 95% CI: 1.000–1.000), AST (*p*=0.001, OR: 1.129, 95% CI: 1.050–1.214), and glucose (*p*=0.008, OR: 1.034, 95% CI: 1.009–1.061) were added significantly to the model (Table 4).

Multinomial logistic regression was used to assess the association between continuous independent laboratory parameters and polychotomous outcomes (NS1, NS1 + IgM, IgM, and dengue negative), with dengue negative as the reference group. In all three outcomes (NS1, both NS1 + IgM, and IgM), thrombocytopenia (*p*=0.006, *p* < 0.001, and *p*=0.001), blood glucose level (*p*=0.029, *p*=0.017, and *p*=0.004), and increased AST (*p*=0.001, *p* < 0.001, and *p* < 0.001) remained common significant parameters while leukopenia was statistically significant only in the NS1 (*p* < 0.001) and both NS1 + IgM (*p* < 0.001) positive groups. In addition, monocytosis (*p*=0.020) was significant in NS1 only positive group while lymphopenia (*p*=0.016) was significant in both NS1 + IgM positive groups (Tables 5 and 6).

### 3.4. Area under the Receiver Operating Characteristics (AUROC) Analysis

Because platelets, glucose, and AST were common in both binary and multinomial logistic regressions, they were regarded as true supportive laboratory parameters; therefore, each parameter was further analyzed for cut-off, sensitivity, and specificity using a ROC curve. Additionally, TLC was also included due to its clinical relevance (Supplementary Figures 2 and 3).

AUC of platelets, TLC, AST, and glucose along with optimal cut-off values that maximize both sensitivity and specificity were analyzed. In short, in the overall analysis, platelets (AUC: 0.924) and AST (AUC: 0.882) demonstrated both sensitivity and specificity above 80%. Among NS1 only, both NS1 + IgM, and IgM only positive participants, both NS1 + IgM positive parameters, i.e., platelets (AUC: 0.832), AST (AUC: 0.803), and TLC (AUC: 0.814), performed better than the other positive subjects, with sensitivity and specificity each above 70%. Although glucose was significant in multivariate logistics, it underperformed TLC in overall (AUC: 0.686 vs. 0.817), in NS1 only (AUC: 0.533 vs. 0.685), and in both NS1 + IgM (AUC: 0.632 vs. 0.814) positive study participants but exceeded it in IgM only positive participants (AUC: 0.712 vs. 0.581) (Table 7).

## 4. Discussion

The major periodic dengue outbreaks in 2010, 2013, 2016, 2019, and now 2022 show a 2–3 year cyclical pattern in Nepal. More sensitive serological tests such as ELISA and dengue confirmatory tests like PCR may not be widely available in developing countries like Nepal. As a result, most settings resort to less sensitive and less specific lateral flow assays for dengue diagnosis. Furthermore, during an outbreak condition, the rapid diagnostic kit is the method of choice due to its feasibility, ease of use, and economic value, as all patients cannot afford many expensive tests. Additionally, in settings where confirmatory tests are not easily accessible, positive rapid tests along with abnormal hematological and biochemical parameters would be essential for proper patient management to prevent life-threatening concerns of dengue.

Our study showed that dengue infection is significantly associated with age, which is also observed in other studies [11, 12]. Moreover, this study showed people in the age group of 20–29 years are more susceptible to infection, which is supported by other studies [13, 14]. Our study also presented the insignificant finding of dengue virus infection with sex, which is in contrast to that reported by Pun et al. [12].

In our study, we analyzed routine hematological and biochemical parameters that may be associated with dengue cases. Among the parameters analyzed, our study demonstrated thrombocytopenia, elevated AST, and increased blood glucose levels to be significantly associated with dengue-positive cases. Likewise, leukopenia, a low lymphocyte count, and monocytosis were significantly associated with certain serological courses of disease.

As per the WHO, hematocrit and thrombocytopenia are the most important laboratory parameters measured during dengue infection [15]. But, our study showed no significant association between hematocrit during the serological course of NS1 and IgM. Few studies report similar findings of insignificance; this may be because our study included only dengue fever patients with mild primary active infections. Thus, there is less chance of plasma leakage, which does not indicate abnormal hematocrit results [16, 17]. Thrombocytopenia, which is well correlated with dengue severity as shown by various studies, also remained significant in our study [18–21]. This decrease in platelets may be due to low production or increased destruction of platelets via activation of the complement factor C_3_ and further binding of the C_5_b-9 complex to the platelet surface [22].

In our study, the median increase in ALT, AST, and ALP was significantly associated with the dengue-positive group; nonetheless, only AST was found to be independently associated. This finding corresponds to studies supporting higher transaminase levels during dengue positivity [23, 24]. ALT is primarily of hepatic origin, while AST is of both hepatic and nonhepatic origin; hence, damage to nonhepatic tissues can also elevate AST as compared to ALT [25]. As a result, despite the significant results obtained in this study, a higher level of AST may not correctly represent hepatic involvement in the dengue-positive group [26]. Furthermore, the recommended drug to minimize dengue symptoms is acetaminophen, which even at therapeutic dosage can cause a temporary elevation in transaminase levels [27, 28].

One study demonstrated that hyperglycemic stress facilitates dengue virus translation and increases protein expression [29]. Our study also showed patients with dengue virus infection had higher glucose levels than the dengue-negative group. A similar outcome has also been presented in a study conducted by Hasanat et al. [30]. Another study suggested prioritizing patients with diabetes mellitus in the diagnosis of dengue but not using it as a factor in assessing dengue severity [31].

In addition to decreased platelets, increased AST levels, and high glucose levels as independent predictors of dengue virus infection, leukopenia with monocytosis (in NS1 only) and leukopenia with lymphopenia (in both NS1 + IgM) were also observed in certain serological durations of illness. Leukopenia with monocytosis and leukopenia with lymphopenia have also been observed in other studies [17, 32, 33]. Virus-induced destruction of WBC and inhibition of myeloid progenitor cells causes leukopenia, while monocytes phagocytose and present the antigen to T- helper cells, causing monocytosis; this explains leukopenia with monocytosis [34, 35].

## 5. Limitation

More sensitive tests such as ELISA and RT-PCR were not available for confirmation of dengue infection. Among the various disadvantages of ICT-based rapid tests is increased crossreactivity, which can lead to false positive outcomes. Furthermore, additional clinical features and disease severity of the patients were not evaluated.

## 6. Conclusion

The study found that certain hematological and biochemical parameters can predict the outcome of dengue infection, which can assist physicians in the diagnosis and proper patient management. Parameters, such as thrombocytopenia, AST, hyperglycemia, and leukopenia with monocytosis (in the NS1-only phase); thrombocytopenia, elevated AST, and high blood glucose (in the IgM-only phase); and thrombocytopenia, elevated AST, high blood glucose, and leukopenia with lymphopenia (in the dual-positive/both NS1 + IgM phase), can provide insight into dengue positivity and help with patient management.

## Figures and Tables

**Table 1:** Hematological and biochemical findings in study participants.

Parameters	Dengue negative (*n* = 171)	Dengue positive (*n* = 177)	*p* value^†^
	Median (*Q*_3_–*Q*_1_)	Median (*Q*_3_–*Q*_1_)	
Age (years)	30.0 (45.0–21.0)	37.0 (55.0–24.0)	**0.006**
Hemoglobin (gm/dl)	13.8 (15.03–13.35)	14.4 (15.67–12.89)	ns
RBC (X 10^12^/L)	4.65 (5.01–4.31)	4.79 (5.19–4.43)	ns
HCT (%)	40.0 (43.5–37.9)	40.7 (44.15–36.65)	ns
MCV (fl)	86.0 (89.0–83.0)	84.9 (88.25–81.3)	**0.041**
MCH (pg)	30.1 (31–28.8)	30.0 (82.55–31.30)	ns
MCHC (gm/dl)	35 (35.2–34.0)	35.5 (36–35.2)	**<0.001**
TLC (cells per *µ*l)	6500 (7500–5370)	3880 (5790–2950)	**<0.001**
Neutrophil (%)	64 (69–58)	67 (76–59)	ns
Lymphocyte (%)	27 (32–23)	21 (30–14)	ns
Monocyte (%)	7 (8–5)	9 (13–7)	**<0.001**
Eosinophil (%)	2 (3–1)	1 (2–1)	**<0.001**
Platelets (per *µ*l)	286000 (336000–223000)	164000 (192000–127000)	**<0.001**
NLR	2.37 (2.91–1.8)	3.37 (5.61–1.96)	ns
LMR	4.29 (5.5–3.5)	2.57 (3.78–1.46)	**<0.001**
Glucose (mg/dl)	87.66 (111.24–73.08)	98.1 (117–86.0)	**<0.001**
Urea (mg/dl)	23.34 (31.26–20.74)	21.78 (27.24–17.1)	ns
Creatinine (mg/dl)	0.84 (0.95–0.73)	0.92 (1.17–0.76)	ns
Total bilirubin (mg/dl)	0.48 (0.58–0.45)	0.50 (0.65–0.40)	**0.009**
Direct bilirubin (mg/dl)	0.11 (0.14–0.05)	0.10 (0.12–0.86)	ns
Total protein (gm/dl)	6.9 (7.3–6.4)	7.06 (7.38–6.65)	**0.006**
Albumin (gm/dl)	4.2 (4.8–3.9)	4.03 (4.32–3.7)	**<0.001**
ALP (U/L)	145 (169–105)	73.77 (129.6–61.07)	**<0.001**
AST (U/L)	25 (28–21)	37.51 (56.23–28.06)	**<0.001**
ALT (U/L)	29 (38–24)	30.64 (48.7–17.47)	**<0.001**
AST/ALT ratio	0.82 (0.93–0.72)	1.29 (1.73–1.02)	**<0.001**

RBC, red blood cell; HCT, hematocrit; MCV, mean cell volume; MCH, mean cell hemoglobin; MCHC, mean cell hemoglobin concentration; TLC, total leukocyte count; NLR, neutrophil lymphocyte ratio; LMR, lymphocyte monocyte ratio; ALP, alkaline phosphatase; AST, aspartate aminotransferase; ALT, alanine aminotransferase; ns, nonsignificant; *p* value <0.05 is considered significant. † signifies analysis via Mann–Whitney *U* test. The bold numerical values in the table represent statistical significance.

**Table 2:** Comparison of hematological and biochemical findings in NS1, IgM, and NS1 + IgM positive dengue patients.

Parameters	Dengue positive (*n* = 177)		
	NS1 only (*n* = 83)	Both NS1 + IgM (*n* = 46)	IgM only (*n* = 48)
	Median (*Q*_3_–*Q*_1_)	Median (*Q*_3_–*Q*_1_)	Median (*Q*_3_–*Q*_1_)
Hemoglobin (gm/dl)	14.66 (15.87–12.90)	14.32 (15.90–12.97)	14.1 (14.7–12.62)
RBC (X 10^12^/L)	4.79 (5.3–4.24)	4.72 (5.19–4.49)	4.82 (5.09–4.42)
HCT (%)	41.4 (44.9–35.9)	40.4 (45.42–37.7)	40.65 (42.5–36.25)
MCV (fl)	86.5 (88.8–83.3)	84.5 (88.12–80.2)	83.7 (87.25–79.7)
MCH (pg)	30.7 (31.8–29.7)	30.0 (31.32–28.2)	29.25 (30.0–27.52)
MCHC (gm/dl)	35.5 (36–35.2)	35.2 (35.82–3.5)	34.75 (35.5–33.9)
TLC (cells per *µ*l)	3880 (5790–2950)	3345 (4442–2497)	4800 (6525–3072)
Neutrophil (%)	67 (76–59)	56.5 (67.75–46)	55 (69–44.25)
Lymphocyte (%)	21 (30–14)	33 (40–22)	32.5 (46–20.25)
Monocyte (%)	9 (13–7)	9.5 (12.25–6)	7 (10.75–5.0)
Eosinophil (%)	1 (2–1)	2 (3–1)	1 (2–1)
Platelets (per *µ*l)	164000 (192000–127000)	113000 (153500–87000)	131000 (177500–53500)
NLR	3.36 (5.61–1.97)	1.83 (3.04–1.12)	1.75 (3.5–1.00)
LMR	2.57 (3.77–1.46)	3.25 (4.23–2.57)	3.9 (6.12–2.34)
Glucose (mg/dl)	98.1 (117–86.0)	104.5 (144.9–91.0)	122 (173–90.9)
Urea (mg/dl)	21.78 (27.24–17.1)	22.13 (31–17.64)	30.04 (37.05–22.75)
Creatinine (mg/dl)	0.92 (1.17–0.76)	0.82 (0.97–0.64)	0.90 (0.99–0.72)
Total bilirubin (mg/dl)	0.49 (0.64–0.4)	0.52 (0.7–0.4)	0.51 (0.66–0.4)
Direct bilirubin (mg/dl)	0.1 (0.12–0.865)	0.1 (0.2–0.08)	0.1 (0.15–0.865)
Total protein (gm/dl)	7.06 (7.38–6.65)	7.24 (7.56–6.89)	6.95 (7.9–6.55)
Albumin (gm/dl)	4.03 (4.31–3.7)	4.1 (4.31–3.86)	4.1 (4.31–3.66)
ALP (U/L)	73.77 (129.6–61.07)	106.4 (207.3–69.28)	139.5 (192.5–90.72)
AST (U/L)	37.51 (56.23–28.06)	76.27 (119.9–44.46)	113 (197–36.75)
ALT (U/L)	30.64 (48.7–17.47)	62.21 (98.9–36.0)	92.42 (156.75–43.5)
AST/ALT ratio	1.29 (1.73–1.02)	1.23 (1.58–1.06)	1.17 (1.48–0.95)

RBC, red blood cell; HCT, hematocrit; MCV, mean cell volume; MCH, mean cell hemoglobin; MCHC, mean cell hemoglobin concentration; TLC, total leukocyte count; NLR, neutrophil lymphocyte ratio; LMR, lymphocyte monocyte ratio; ALP, alkaline phosphatase; AST, aspartate aminotransferase; ALT, alanine aminotransferase.

**Table 3:** Kruskal–Wallis test and the post hoc Dunn's pairwise analysis with Bonferroni adjustment among the dengue negative group and NS1 positive, IgM positive, and both NS1 + IgM positive dengue patients.

Parameters	Kruskal–Wallis test		Dunn's pairwise comparison with Bonferroni adjustment					
	Dengue positive (NS1, NS1 + IgM (B), IgM)		*p* value					
	Dengue negative (C)							
	*H*	*p* value	NS1/C	B/C	IgM/C	B/IgM	B/NS1	NS1/IgM
Age (yrs)	7.949	**0.047**	ns	ns	ns	ns	ns	ns
Hemoglobin (gm/dl)	7.491	ns	—	—	—	—	—	—
RBC (X 10^12^/L)	2.756	ns	—	—	—	—	—	—
HCT (%)	2.923	ns	—	—	—	—	—	—
MCV (fl)	11.721	**0.008**	ns	ns	**0.013**	ns	ns	**0.044**
MCH (pg)	21.988	**<0.001**	ns	ns	**0.04**	ns	ns	**<0.001**
MCHC (gm/dl)	54.099	**<0.001**	**<0.001**	**0.024**	ns	ns	ns	**<0.001**
TLC (cells per *µ*l)	116.280	**<0.001**	**<0.001**	**<0.001**	**<0.001**	**0.004**	ns	ns
Neutrophil (%)	22.970	**<0.001**	ns	**0.035**	ns	ns	**<0.001**	**<0.001**
Lymphocyte (%)	29.998	**<0.001**	**<0.001**	ns	ns	ns	**<0.001**	**<0.001**
Monocyte (%)	47.874	**<0.001**	**<0.001**	**<0.001**	ns	ns	ns	ns
Eosinophil (%)	51.000	**<0.001**	**<0.001**	ns	**<0.001**	ns	**0.002**	ns
Platelets (per *µl*)	193.996	**<0.001**	**<0.001**	**<0.001**	**<0.001**	ns	ns	ns
NLR	28.772	**<0.001**	**0.002**	ns	ns	ns	**<0.001**	**<0.001**
LMR	65.861	**<0.001**	**<0.001**	**0.001**	ns	ns	ns	**<0.001**
Glucose (mg/dl)	45.213	**<0.001**	**0.012**	**<0.001**	**<0.001**	ns	ns	**0.016**
Urea (mg/dl)	17.610	**0.001**	ns	**0.035**	ns	**0.012**	ns	**<0.001**
Creatinine (mg/dl)	10.893	**0.012**	**0.016**	ns	ns	**0.046**	ns	ns
Total bilirubin (mg/dl)	7.939	**0.047**	ns	ns	ns	ns	ns	ns
Direct bilirubin (mg/dl)	2.015	ns	—	—	—	—	—	—
Total protein (gm/dl)	12.869	**0.005**	ns	0.003	ns	ns	ns	ns
Albumin (gm/dl)	1.859	**0.002**	**0.002**	ns	ns	ns	ns	ns
ALP (U/L)	38.872	**<0.001**	**<0.001**	ns	ns	ns	**0.011**	**<0.001**
AST (U/L)	166.520	**<0.001**	**<0.001**	**<0.001**	**<0.001**	ns	**0.013**	**0.008**
ALT (U/L)	80.633	**<0.001**	ns	**<0.001**	**<0.001**	**<0.001**	ns	**<0.001**
AST/ALT ratio	105.156	**<0.001**	**<0.001**	**<0.001**	**<0.001**	ns	ns	ns

RBC, red blood bell; HCT, hematocrit; MCV, mean cell volume; MCH, mean cell hemoglobin; MCHC, mean cell hemoglobin concentration; TLC, total leukocyte count; NLR neutrophil lymphocyte ratio; LMR, lymphocyte monocyte ratio; ALP, alkaline phosphatase; AST, aspartate aminotransferase; ALT, alanine aminotransferase; ns, nonsignificant; *p* < 0.05 is considered significant. The bold numerical values in the table represent statistical significance.

**Table 4:** Binary logistic regression analysis for laboratory parameters in overall dengue-positive study participants.

Parameter	Univariate analysis		Multivariate analysis	
	OR_C_ (95% CI)	*p* value	OR_A_ (95% CI)	*p* value
MCHC	1.615 (1.290–2.023)	**<0.001**	ns	ns
Platelet	1.000 (1.000–1.000)	**<0.001**	1.000 (1.000–1.000)	**<0.001**
TLC	0.999 (0.999–1.000)	**<0.001**	1.000 (0.999–1.000)	**0.002**
Neutrophil	0.983 (0.966 1.001)	**0.064**	ns	ns
Lymphocyte	1.003 (0.982–1.023)	0.804	—	—
Monocytes	1.319 (1.213–1.434)	**<0.001**	ns	ns
LMR	0.879 (0.798–0.968)	**0.009**	ns	ns
Glucose	1.026 (1.017–1.035)	**<0.001**	1.034 (1.009–1.061)	**0.008**
Total protein	1.584 (1.080–2.325)	**0.019**	ns	ns
Albumin	0.505 (0.326–0.780)	**0.002**	ns	ns
ALT	1.044 (1.030–1.059)	**<0.001**	ns	ns
AST	1.159 (1.113–1.207)	**<0.001**	1.129 (1.050–1.214)	**0.001**
ALP	0.997 (0.993–1.000)	**0.056**	ns	ns
AST/ALT	18.902 (8.759–40.793)	**<0.001**	ns	ns

RBC, red blood cell; HCT, hematocrit; MCV, mean cell volume; MCH, mean cell hemoglobin; MCHC, mean cell hemoglobin concentration; TLC, total leukocyte count; NLR, neutrophil lymphocyte ratio; LMR, lymphocyte monocyte ratio; ALP, alkaline phosphatase; AST, aspartate aminotransferase; ALT, alanine aminotransferase; OR_C_, crude odds ratio; OR_A_, adjusted odds ratio. In univariate logistics, *p* < 0.25 was considered statistically significant. In multivariate analysis, *p* < 0.05 was considered statistically significant. The bold numerical values in the table represent statistical significance.

**Table 5:** Univariate logistic regression analysis for laboratory parameters in the NS1-only, both NS1 + IgM, and IgM-only positive dengue patients.

Univariate logistics						
Parameters	NS1 only		Both NS1 + IgM		IgM only	
	OR_C_ (95% CI)	*p* value	OR_C_ (95% CI)	*p* value	OR_C_ (95% CI)	*p* value
MCHC	2.768 (1.943–3.943)	**<0.001**	1.464 (1.028–2.085)	**0.035**	0.997 (0.732–1.357)	0.983
Platelet	1.000 (1.000–1.000)	**<0.001**	1.000 (1.000–1.000)	**<0.001**	1.000 (1.000–1.000)	**<0.001**
TLC	0.999 (0.999–1.000)	**<0.001**	0.999 (0.999–0.999)	**<0.001**	1.000 (1.000–1.000)	**0.022**
Neutrophil	1.023 (0.998–1.049)	**0.075**	0.949 (0.924–0.976)	**<0.001**	0.956 (0.930–0.983)	**0.001**
Lymphocyte	0.945 (0.916–0.975)	**<0.001**	1.039 (1.007–1.072)	**0.017**	1.049 (1.018–1.082)	**0.002**
Monocytes	1.346 (1.225–1.479)	**<0.001**	1.389 (1.250–2.543)	**<0.001**	1.218 (1.093–1.358)	**<0.001**
LMR	0.5240 (0.426–0.645)	**<0.001**	0.846 (0.715–1.002)	**0.052**	1.044 (0.969–1.126)	0.256
Glucose	1.019 (1.009–1.029)	**<0.001**	1.033 (1.023–1.044)	**<0.001**	1.034 (1.023–1.045	**<0.001**
Total protein	1.415 (0.878–2.279)	**0.154**	2.645 (1.434–4.879)	**0.002**	1.209 (0.676–2.159)	0.522
Albumin	0.464 (0.268–0.806)	**0.006**	0.645 (0.338–1.232)	**0.184**	0.451 (0.229–0.885)	**0.021**
ALT	1.035 (1.020–1.051)	**<0.001**	1.053 (1.037–1.069)	**<0.001**	1.055 (1.039–1.071)	**<0.001**
AST	1.153 (1.108–1.201)	**<0.001**	1.168 (1.121–1.216)	**<0.001**	1.169 (1.123–1.218)	**<0.001**
ALP	0.988 (0.982–0.993)	**<0.001**	1.002 (0.997–1.007)	0.552	1.002 (0.998–1.007)	0.323
AST/ALT	21.338 (9.386–8.510)	**<0.001**	21.221 (8.782–51.280)	**<0.001**	14.106 (5.777–34.006)	**<0.001**

RBC, red blood cell; HCT, hematocrit; MCV, mean cell volume; MCH, mean cell hemoglobin; MCHC, mean cell hemoglobin concentration; TLC, total leukocyte count; NLR, neutrophil lymphocyte ratio; LMR, lymphocyte monocyte ratio; ALP, alkaline phosphatase; AST, aspartate aminotransferase; ALT, alanine aminotransferase; OR_C_, crude odds ratio; OR_A_, adjusted odds ratio; *p* < 0.25 was considered statistically significant. The bold numerical values in the table represent statistical significance.

**Table 6:** Multinomial multivariate logistic regression analysis for laboratory parameters in NS1, both NS1 + IgM, and IgM positive dengue patients with the dengue negative group as reference.

Multivariate logistics						
	NS1 only		Both NS1 + IgM		IgM only	
Parameters	OR_A_ (95% CI)	*p* value	OR_A_ (95% CI)	*p* value	OR_A_ (95% CI)	*p* value
MCHC	ns	ns	ns	ns	—	—
Platelets	1.000 (1.000–1.000)	**0.006**	1.000 (1.000–1.000)	**<0.001**	1.000 (1.000–1.000)	**0.001**
TLC	0.999 (0.999–1.000)	**<0.001**	0.999 (0.999–1.000)	**<0.001**	ns	ns
Neutrophil	ns	ns	ns	ns	ns	ns
Lymphocyte	ns	ns	0.520 (0.306–0.884)	**0.016**	ns	ns
Monocyte	2.332 (1.140–4.771)	**0.020**	ns	ns	ns	ns
LMR	ns	ns	ns	ns	—	—
Glucose	1.028 (1.003–1.055)	**0.029**	1.031 (1.006–1.057)	**0.017**	1.037 (1.012–1.062)	**0.004**
Total protein	ns	ns	ns	ns	—	—
Albumin	ns	ns	ns	ns	ns	ns
ALT	ns	ns	ns	ns	ns	ns
AST	1.131 (1.050–1.218)	**0.001**	1.136 (1.056–1.222)	**<0.001**	1.141 (1.063–1.225)	**<0.001**
ALP	ns	ns	—	—	—	—
AST/ALT	ns	ns	ns	ns	ns	ns

RBC, red blood cell; HCT, hematocrit; MCV, mean cell volume; MCH, mean cell hemoglobin; MCHC, mean cell hemoglobin concentration; TLC, total leukocyte count; NLR neutrophil lymphocyte ratio; LMR, lymphocyte monocyte ratio; ALP, alkaline phosphatase; AST, aspartate aminotransferase; ALT, alanine aminotransferase; OR_C_, crude odds ratio; OR_A_, adjusted odds ratio; *p* <  0.05 was considered statistically significant. The bold numerical values in the table represent statistical significance.

**Table 7:** AUROC of laboratory parameters in overall positive, NS1 only, NS1 + IgM, and IgM only-positive dengue patients.

Dengue positive	Parameters	AUC	S.E.	*p*value	95% CI	Cut-off	Sensitivity (%)	Specificity (%)
Overall	Platelets	0.924	0.014	<0.001	0.897–0.951	200000	83.6	83.6
	TLC	0.817	0.023	<0.001	0.771–0.862	5440	75.1	74.9
	AST	0.882	0.019	<0.001	0.845–0.919	29.21	80.8	83.0
	Glucose	0.686	0.028	<0.001	0.630–0.741	95.16	62.7	62.6

NS1 only	Platelets	0.701	0.027	<0.001	0.648–0.755	179000	65.1	65.7
	TLC	0.685	0.035	<0.001	0.617–0.752	4995	63.9	63.8
	AST	0.631	0.032	<0.001	0.568–0.694	32.30	63.9	63.4
	Glucose	0.533	0.035	0.357	0.465–0.602	96.75	53.0	53.6

Both NS1 + IgM	Platelets	0.832	0.026	<0.001	0.781–0.884	154000	76.1	76.8
	TLC	0.814	0.030	<0.001	0.754–0.874	4304	73.9	75.8
	AST	0.803	0.031	<0.001	0.743–0.863	43.21	76.1	76.2
	Glucose	0.632	0.046	0.004	0.542–0.721	101.43	58.7	58.6

IgM only	Platelets	0.764	0.038	<0.001	0.688–0.839	166500	70.8	71.0
	TLC	0.581	0.046	0.073	0.491–0.670	4995	58.3	59.7
	AST	0.811	0.038	<0.001	0.735–0.886	39.43	75.0	75.0
	Glucose	0.712	0.044	<0.001	0.625–0.788	103.25	62.5	62.7

TLC, total leukocyte count; AST, aspartate aminotransferase; AUC was interpreted as follows: 0.5 to 0.6, unsatisfactory; 0.6 to 0.7, satisfactory; 0.7 to 0.8, good; 0.8 to 0.9, very good; and >0.9, excellent.

## Data Availability

The datasets of the current study are available from the corresponding author upon reasonable request.

## Abbreviations

ALP: Alkaline phosphatase
ALT: Alanine aminotransferase
AST: Aspartate aminotransferase
AUC: Area under curve
CI: Confidence interval
DENV: Dengue virus
Hb: Hemoglobin
ICT: Immunochromatography
IgG: Immunoglobulin G
IgM: Immunoglobulin M
LMR: Lymphocyte monocyte ratio
MCH: Mean cell hemoglobin
MCHC: Mean cell hemoglobin concentration
MCV: Mean cell volume
NLR: Neutrophil lymphocyte ratio
NS1: Nonstructural protein 1
OR_A_: Adjusted odds ratio
OR_C_: Crude odds ratio
RBC: Red blood cell
ROC: Receiver operating characteristics
WBC: White blood cell.

## Supplementary Materials

Supplementary Materials Supplementary Figure 1 represents the distribution pyramid of dengue-positive cases according to age group and gender. From the figure, we can observe that males were predominantly infected (*n* = 111; 62.7%) more than females (*n* = 66; 37.3%). Moreover, the age group of 20–29 years was found to be more susceptible to infection in both genders. Supplementary Figure 2 demonstrates the ROC of platelets and the total leukocyte count (TC) of dengue-positive cases. The green line in the figure represents the diagonal reference point. The closer the diagnostic test (platelets and TC) line is to the reference line, the lower the diagnostic performance of the test is observed. Thus, the authors can observe that platelets and TC performed better in the overall ROC model and both NS1 + IgM positive models than the rest of the models. Supplementary Figure 3 shows the ROC of glucose and aspartate aminotransferase (AST) in dengue-positive cases. The green line in the figure represents the diagonal reference point. The closer the diagnostic test (glucose and AST) line is to the reference line, the low the diagnostic performance of the test is observed. Thus, the authors can observe that AST performed better in the overall ROC and both NS1 + IgM models than the rest of the models, while glucose performed better in the overall ROC model and the IgM-only model.
