# Supplementary figures and images for: Association of Hematological and Biochemical Parameters with Serological Markers of Acute Dengue Infection during the 2022 Dengue Outbreak in Nepal
Source: J Trop Med. 2023 Feb 23;2023:2904422. doi: 10.1155/2023/2904422 (PMC9981284; doi:10.1155/2023/2904422)

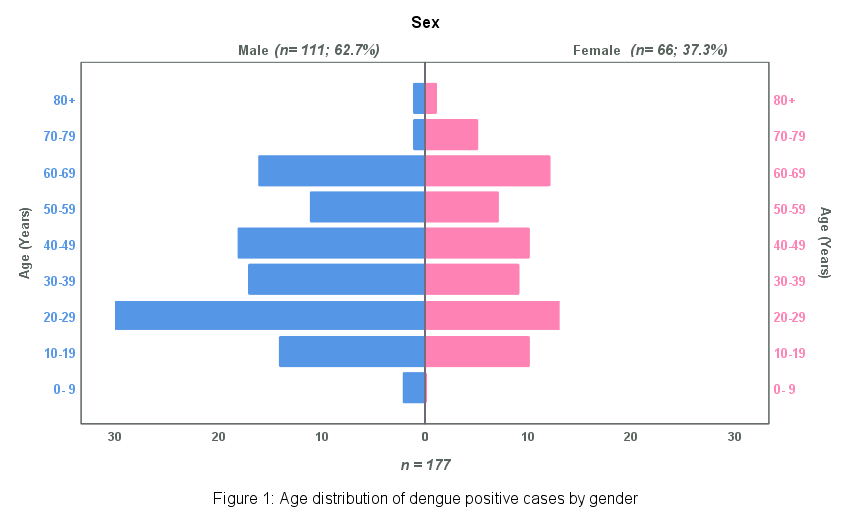


Supplementary Figure 1: Distribution of dengue-positive cases by age group and gender

Supplement: Supplementary Materials — Supplementary Figure 1 represents the distribution pyramid of dengue-positive cases according to age group and gender. From the figure, we can observe that males were predominantly infected (n = 111; 62.7%) more than females (n = 66; 37.3%). Moreover, the age group of 20–29 years was found to be more susceptible to infection in both genders. Supplementary Figure 2 demonstrates the ROC of platelets and the total leukocyte count (TC) of dengue-positive cases. The green line in the figure represents the diagonal reference point. The closer the diagnostic test (platelets and TC) line is to the reference line, the lower the diagnostic performance of the test is observed. Thus, the authors can observe that platelets and TC performed better in the overall ROC model and both NS1 + IgM positive models than the rest of the models. Supplementary Figure 3 shows the ROC of glucose and aspartate aminotransferase (AST) in dengue-positive cases. The green line in the figure represents the diagonal reference point. The closer the diagnostic test (glucose and AST) line is to the reference line, the low the diagnostic performance of the test is observed. Thus, the authors can observe that AST performed better in the overall ROC and both NS1 + IgM models than the rest of the models, while glucose performed better in the overall ROC model and the IgM-only model. [file 2904422.f1.zip › Supplementary Figure 1 Distribution graph of Dengue Positive cases on basis of age and gender.docx]

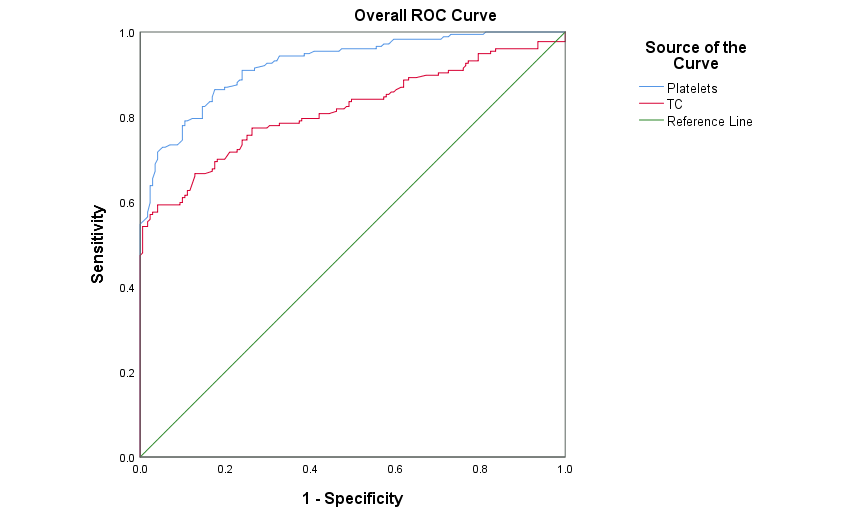

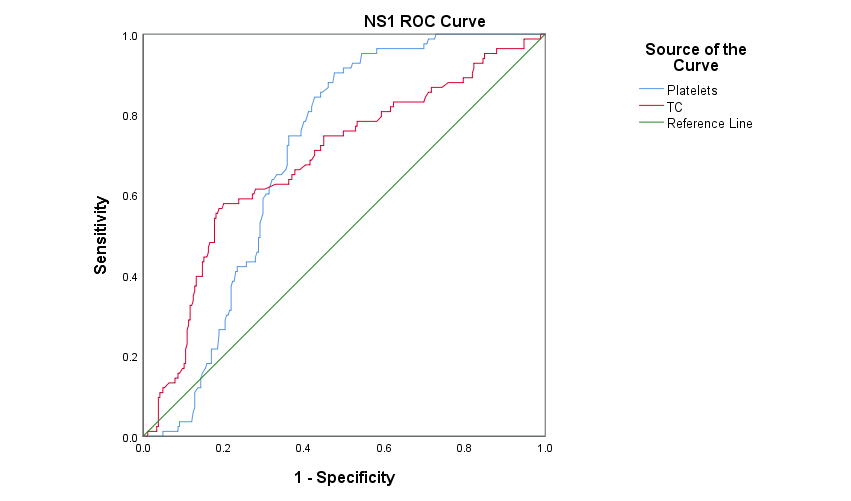

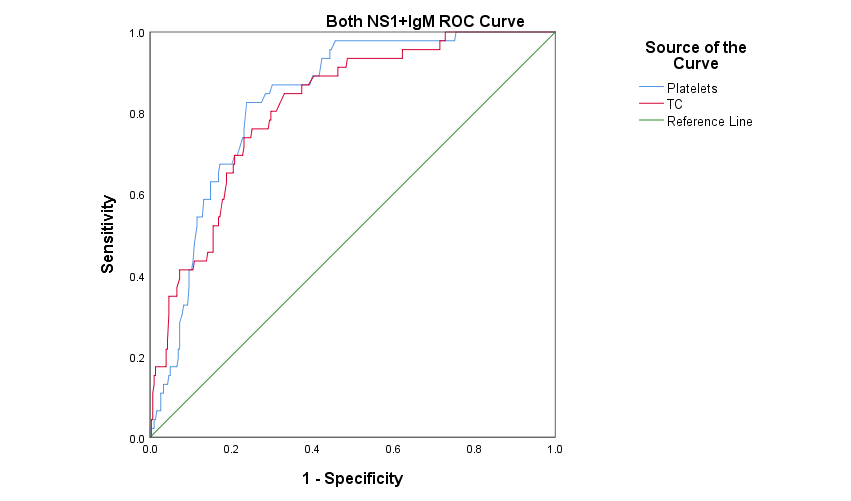

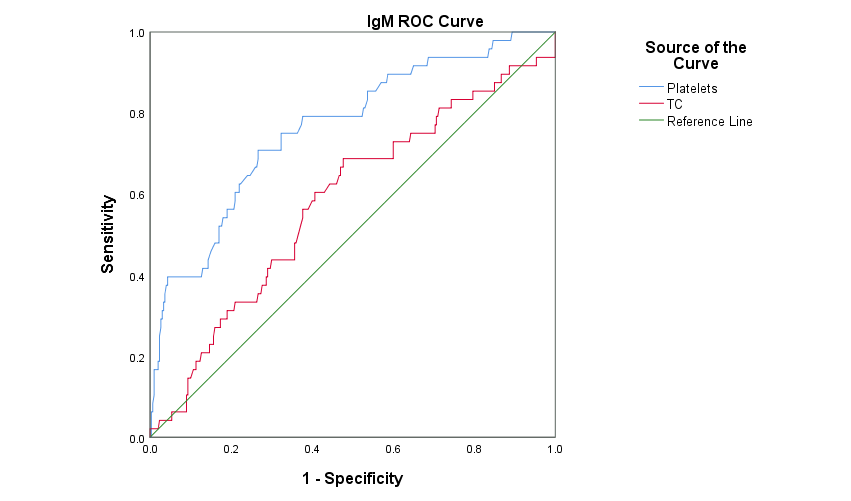


**Supplementary Figure 2: ROC curve of platelets and Total leucocyte count (TC)**

Supplement: Supplementary Materials — Supplementary Figure 1 represents the distribution pyramid of dengue-positive cases according to age group and gender. From the figure, we can observe that males were predominantly infected (n = 111; 62.7%) more than females (n = 66; 37.3%). Moreover, the age group of 20–29 years was found to be more susceptible to infection in both genders. Supplementary Figure 2 demonstrates the ROC of platelets and the total leukocyte count (TC) of dengue-positive cases. The green line in the figure represents the diagonal reference point. The closer the diagnostic test (platelets and TC) line is to the reference line, the lower the diagnostic performance of the test is observed. Thus, the authors can observe that platelets and TC performed better in the overall ROC model and both NS1 + IgM positive models than the rest of the models. Supplementary Figure 3 shows the ROC of glucose and aspartate aminotransferase (AST) in dengue-positive cases. The green line in the figure represents the diagonal reference point. The closer the diagnostic test (glucose and AST) line is to the reference line, the low the diagnostic performance of the test is observed. Thus, the authors can observe that AST performed better in the overall ROC and both NS1 + IgM models than the rest of the models, while glucose performed better in the overall ROC model and the IgM-only model. [file 2904422.f1.zip › Supplementary Figure 2 ROC curve of platelets and Total leukocyte count (TC).docx]

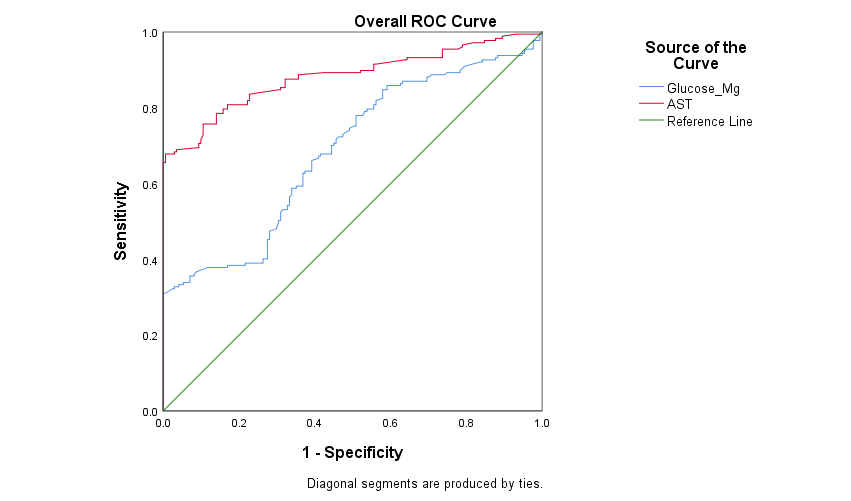

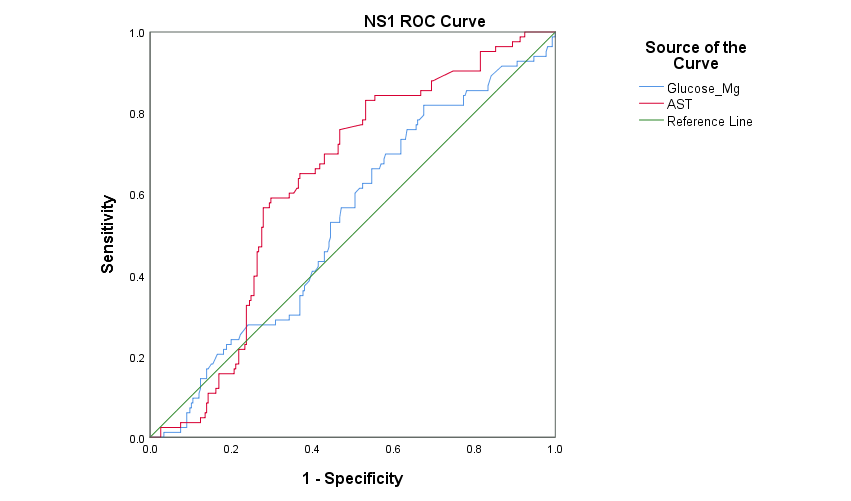

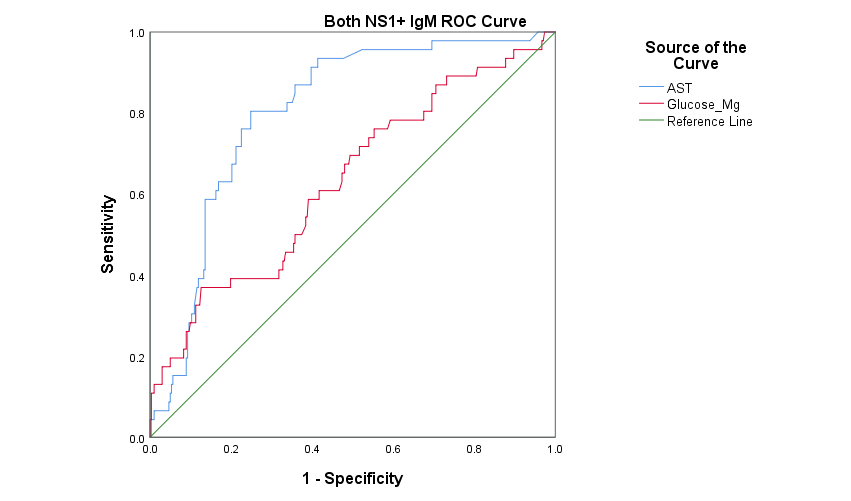

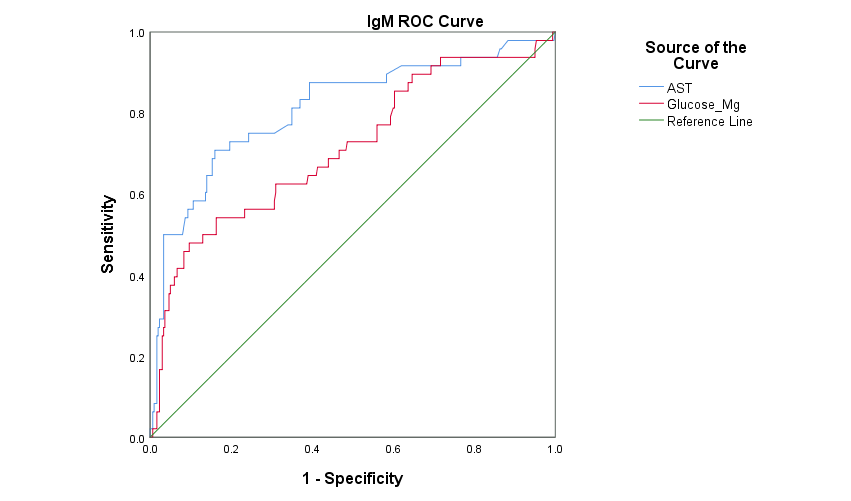


**Supplementary Figure 3: ROC curve of glucose and aspartate aminotransferase (AST)**

Supplement: Supplementary Materials — Supplementary Figure 1 represents the distribution pyramid of dengue-positive cases according to age group and gender. From the figure, we can observe that males were predominantly infected (n = 111; 62.7%) more than females (n = 66; 37.3%). Moreover, the age group of 20–29 years was found to be more susceptible to infection in both genders. Supplementary Figure 2 demonstrates the ROC of platelets and the total leukocyte count (TC) of dengue-positive cases. The green line in the figure represents the diagonal reference point. The closer the diagnostic test (platelets and TC) line is to the reference line, the lower the diagnostic performance of the test is observed. Thus, the authors can observe that platelets and TC performed better in the overall ROC model and both NS1 + IgM positive models than the rest of the models. Supplementary Figure 3 shows the ROC of glucose and aspartate aminotransferase (AST) in dengue-positive cases. The green line in the figure represents the diagonal reference point. The closer the diagnostic test (glucose and AST) line is to the reference line, the low the diagnostic performance of the test is observed. Thus, the authors can observe that AST performed better in the overall ROC and both NS1 + IgM models than the rest of the models, while glucose performed better in the overall ROC model and the IgM-only model. [file 2904422.f1.zip › Supplementary Figure 3 ROC curve of AST and Glucose.docx]
